# Osteogenic potential of nanostructured Cu/W/Co in Fe-Mn alloys designed for maxillofacial applications: in vivo study in dog model

**DOI:** 10.1007/s10856-025-06890-7

**Published:** 2025-06-25

**Authors:** Mohamed A. Abdel Hamid, Samir A. Elborolosy, Sara El Moshy, Hany R. Ammar, S. Sivasankaran, Walid S. Salem, Elham A. Hassan

**Affiliations:** 1 Department of Surgery, Anesthesiology and Radiology- Faculty of Veterinary Medicine- Cairo University, Giza, 12211 Egypt; 2 Oral and Maxillofacial Surgery Department- Faculty of Dentistry- Beni-Suef University, Beni-Suef, Egypt; 3 Department of Mechanical Engineering, College of Engineering, Cairo University, Cairo, 12613 Egypt; 4 Department of Mechanical Engineering, College of Engineering, Qassim University, Buraydah, 51452 Saudi Arabia; 5 Oral and Maxillofacial Radiology Department- Faculty of Dentistry- Beni-Suef University, Beni-Suef, Egypt

## Abstract

To investigate osteogenic potential of biodegradable nanostructured Cu/W/Co in Fe-Mn alloys for maxillofacial applications in an in vivo model. Nanostructured FeMn_35_, FeMn_32_Cu_3_, FeMn_32_W_3,_ FeMn_32_Co_3_ alloys were fabricated. Ten mongrel dogs were included where five mandibular defects were induced in each dog. Defects were randomly allocated into 5 groups ((M) control defects covered by bone disc, (M0) implanted by FeMn_35_ alloy, (M1) implanted by FeMn_32_Cu_3_ alloy, (M2) implanted by FeMn_32_W_3_ alloy, (M3) implanted by FeMn_32_Co_3_ alloy). Dogs were euthanized at 12 weeks for cone beam computed tomography, histologic and immunohistologic evaluation, and gene expression of osteopontin and osteocalcin. Defects implanted with metal demonstrated thicker bone trabeculae mixed with lamellar bone while control defects (M) demonestrated immature woven bone. Quantitative evaluation of bone area %, area % of mature bone and expression of osteopentin and osteocalcin bone markers revealed a statistically significant highest bone area % and maturation in group M3 compared to M2, M1, M0, and M group. A statistically significant increase in bone area % and maturation was recorded in M2 group compared to M1, M0, and M groups. A significantly increased bone area % and maturation was recorded in M1 compared to control M group and also between M0 and M group. Incorporating Cu/W/Co into Fe-Mn alloys enhanced biocompatibility and improved bone regeneration suggesting its suitability for use in various orthopedic and dental applications. Biodegradable metal alloys could improve patient outcome, reduce the need for additional surgeries to remove nonbiodegradble implants. Biodegradable metal alloys could improve patient outcome, reduce the need for additional surgeries to remove nonbiodegradble implants.

**Graphical Abstract:**

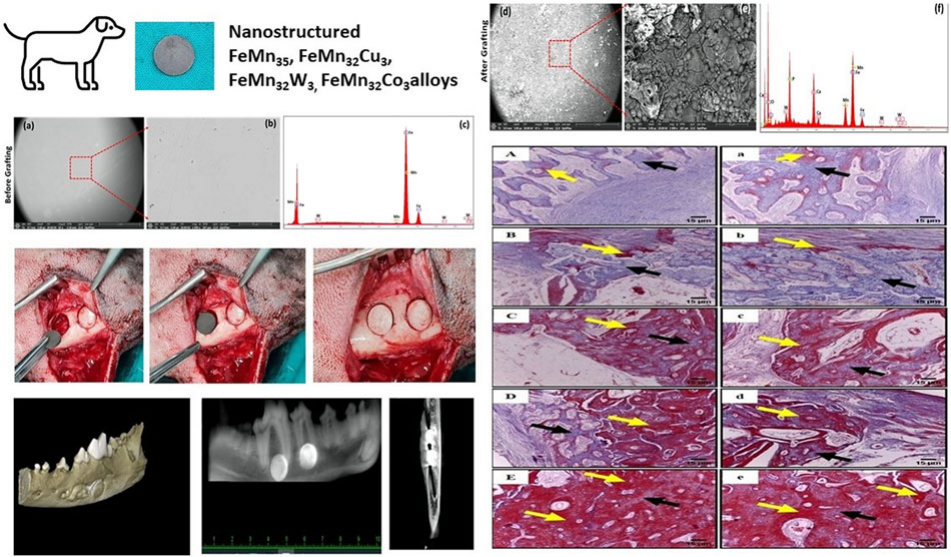

## Introduction

Nanostructured biodegradable alloys have shown great potential as bioactive materials for reconstruction of bone defects caused by traumatic fractures or tumor resection [1]. An alloy is a metallic material formed by blending two or more metals or by combining metals and nonmetals [2]. In the molten state, metals can dissolve in different extents enabling the formation of alloys as they solidify [3]. Non-biodegradable metals, such as titanium and stainless steel, are widely used in maxillofacial surgery due to their excellent mechanical strength and biocompatibility. However, they require additional surgery to remove the implants, causing surgical pain and economic burden [2].

Biodegradable metal alloys (BMAs) have emerged as promising materials to fabricate temporary biomedical implants, with the purpose of avoiding the potential side effects of permanent implants. They have the advantages of being biodegradable with excellent mechanical strength, ductility, formability, osteogenic capacity, and antibacterial properties. BMAs eliminate the need for additional surgery for implant removal, which significantly reduces patient discomfort and, post-operative complications as well as treatment costs [2, 4].

Iron (Fe) and Fe-based biomaterials are one of the earlier BMAs used in maxillofacial surgery. The suitability of Fe as a biodegradable implant has been demonstrated in preliminary in vivo animal models [5, 6]. Fe-implants did not demonstrate any local or systemic toxicity nor significant inflammatory reactions when implanted within the body. Fe alloys are of excellent mechanical strength resembling stainless steels [2, 7]. Potential disadvantages of Fe alloys include the slow degradation rate, release of insoluble degradation products, and ferromagnetic properties [2].

Speeding of the degradation rate of Fe-based implants could be achieved by alloying. An iron-manganese (Fe-Mn) binary composition was introduced with enhanced corrosion rate, biological activity, cell compatibility and reduced ferromagnetic properties [4].

Copper (Cu) alloys have gained popularity in maxillofacial surgery due to their excellent combination of mechanical properties and biocompatibility. Cu has been combined with titanium, nickel and zinc in various orthodontic brackets and dental implants [8]. One of the key features of Cu alloys is their high strength, corrosion resistance, reduced allergic reaction with antimicrobial properties, which help to prevent infection at surgical site and reduce post-operative complications [9].

Tungsten (W) is a naturally occurring element used in industry and medical devices. Tungsten accumulates in bone and still, there is little known about the role of tungsten as a modulator of bone homeostasis. It has been suggested that the predominant form of tungsten in bone may not be sodium tungstate but rather phosphotungstate, suggesting that tungsten may integrate itself as part of the inorganic hydroxyapatite component of bone [10]. The incorporation of tungsten into the bone matrix may explain its slow rate of release from bone [11]. Osteoclast activity could modulate the form of tungsten within the bone. Tungsten alone was insufficient to promote osteoclastogenesis [12]. In vitro, tungsten decreases mesenchymal stem cell differentiation toward osteoblasts, and osteoblastic gene expression of the master transcription factor Runx2 along with downstream targets Sp7 (Osterix) and Bglap (Osteocalcin) [13]. Thus, tungsten may alter both osteoblasts and osteoclasts. Tungsten enhances adipogenesis of mesenchymal stem cells both in vitro and in vivo [13].

Cobalt is a trace element required for normal biological functions. The normal level of cobalt in the human body ranges between 1.1–1.5 mg, with 43% present in muscles, 14% in bone, and the remaining percent in soft tissues [14]. Studies indicated that incorporating cobalt into bioactive glass and hydroxyapatite has activated bone marrow stem cell and osteoblast proliferation, VEGF secretion, as well as the up-regulation of bone-related genes [15]. Cobalt ions can promote a hypoxia-like response and stimulates angiogenesis with osteogenesis [15, 16].

To the authors’ knowledge, the incorporation of Cu, W, and Co into the Fe-Mn alloy in an in vivo study was not previously reported.The aim of the present investigation was to study the osteogenic properties of nanostructured Cu/W/Co in Fe-Mn alloys designed for maxillofacial applications in an in vivo dog model.

## Materials and methods

### Material preparation

High purity (99.99%) Metals powders (Fe, Mn, Cu, W, and Co) were used to synthesis the biodegradable alloys. These elemental powders were mixed in atomic weight percent to produce four biodegradable alloys: FeMn_35_, FeMn_32_Cu_3_, FeMn_32_W_3_, and FeMn_32_Co_3_. The elemental powders were mechanically alloyed in a Pulverisette 5/2 planetary ball mill for 5 h with 300 rpm, ball-to-powder mass ratio of 10:1. The milling was performed in toluene, as process control agent. The developed alloys in the powders form were subjected to a stress relief heat treatment under vacuum in a tube furnace at 150 °C for 1 h. The heat-treated powders were then consolidated into dense solid samples by selective laser melting process. Detailed description of alloy fabrication, characterization, microstructure, mechanical performance and corrosion behavior was published separately [17, 18].

Solid samples of 15 mm diameter were fabricated and sectioned into a 1 mm thickness discs to be used for in vivo implantation.

### Microstructural characterization

Apreo field emission gun high resolution scanning electron microscope (Apreo FEG-HR-SEM, 30 keV, 1.3 nm resolution at 1 keV) equipped with backscattered electron (BSE), and energy dispersive x-ray (EDX) detectors was used to investigate the elemental analysis of the developed alloy samples before and after in vivo application. The EDX system was operated by TEAM software for data acquisition and spectrum analysis. The mean weight percent (Wt%) and atomic percent (At%) were calculated for each element. The error percent (Er. %) was calculated to document the uncertainty in the statistical confidence in elements quantification.

### In vivo dog model

#### Animals

An experimental study included ten skeletally mature male mongrel dogs weighing 23.7 ± 1.9 kg, aging 19.9 ± 2.7 months. Clinical and hematological examination was performed for each dog to exclude the evidence of systemic or bone disease. Dogs were quarantined for two weeks and received anti-parasitic medication. During the study, dogs were housed individually in separate cages, fed twice daily with free access to drinking water. All study procedures comply with the ARRIVE guidelines and were approved by the Institutional Animal Care and Use Ethical Committee of Faculty of Veterinary Medicine- Cairo University (Approval # Vet CU 01122022549).

### Study design

A randomized controlled experimental study was designed where five surgically induced cylindrical critical-sized (15 mm diameter × 4 mm depth) mandibular defects were created into the right and left side of the mandible using a 15 mm diameter trephine bur (5 defects/dog). Bone defects were randomly allocated into one of the following five groups:

- **Control (M) group:** defects were created and left empty.
- **FeMn**_**35**_ **(M0) group:** defects were implanted by the biodegradable FeMn_35_ alloy_._
- **FeMn**_**32**_**Cu**_**3**_ **(M1) group:** defects were implanted by the biodegradable FeMn_32_Cu_3_ alloy_._
- **FeMn**_**32**_**W**_**3**_ **(M2) group:** defects were implanted by the biodegradable FeMn_32_W_3_ alloy_._
- **FeMn**_**32**_**Co**_**3**_ **(M3) group:** defects were implanted by the biodegradable FeMn_32_Co_3_ alloy_._

**Fig. 1:**
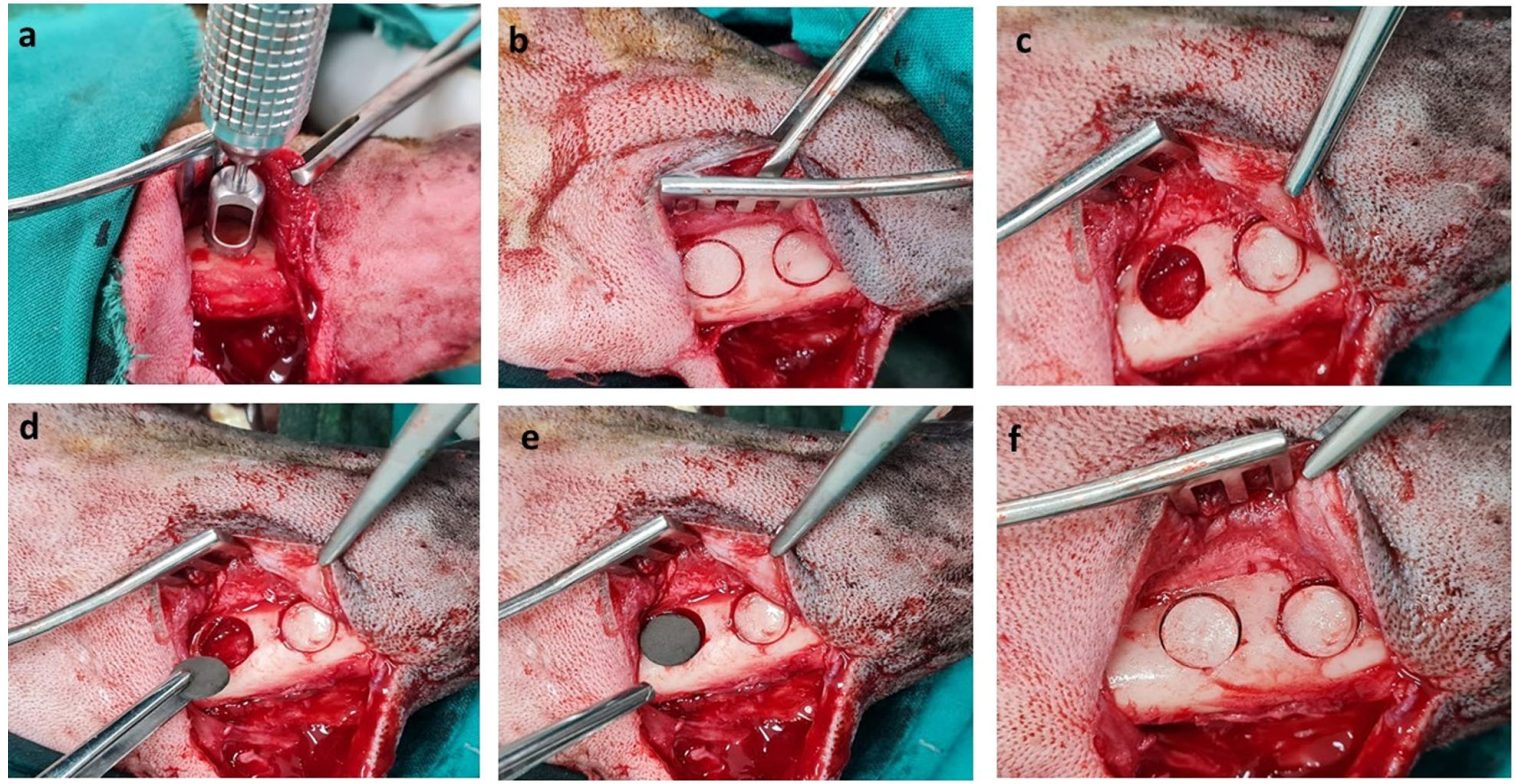
Intra-operative radiograph demonstrating induction of a 15-mm circular mandibular defect using a trephine bur (**a**–**c**). Implantation of the biodegradable metal alloys based on defect grouping (**d**, **e**). Bone defects were covered by the preserved bone discs that were press fitted over the implanted alloys (**f**)

In all groups, bone defects with the implanted alloy were covered by the preserved bone disc while maintaining good alignment with the surrounding bones (Fig. 1).

Dogs were monitored daily and evaluated clinically till 12 weeks, where dogs were euthanized for Cone beam computed tomography (CBCT) and histologic and histomorphometric evaluation.

#### Anesthetic protocol

The cephalic vein was cannulated and the skin over the right and left mandibles was prepared for aseptic surgery. Dogs were tranquilized with intramuscular xylazine HCL 2% (Xylaject®, ADWIA Co. Egypt) at a dose of 1 mg/kg. Atropine sulphate 0.1% (Atropine Sulfate®, El Nasr pharm. Chem. Co. Egypt) was given subcutaneously 15 min before anaesthesia. Induction of anesthesia was made through ketamine HCL 5% (Ketamine®, Rotex Medica, Germany) at a dose of 10 mg/kg. Anesthesia was maintained by thiopental sodium 2.5% (Anapental®: Sigma-Tec, Egypt) that was injected intravenously at a dosage of 25 mg/kg.

#### Implantation of the biodegradable alloys

Using an extra-oral approach, a 6-cm skin incision was made 1 cm below the inferior border of the mandible to visualize mandibular body. The deep fascia was dissected, and the periosteum was incised horizontally and elevated. A circular mandibular defect (15 mm diameter) was created using a trephine bur (Trephine drill, Friatec AG, Germany). Saline irrigation was maintained to avoid heat necrosis, the inferior alveolar canal was carefully avoided during drilling. Care was taken to maintain the integrity of the bone disc covering the induced defects. Biodegradable metal alloys were inserted into defects based on defect grouping. The preserved bone discs were press fitted over the implanted alloys (Fig. 1). Routine closure of the subcutaneous and cutaneous incision was made using 2-0 Vicryl suture.

#### Post-operative care and follow up

Daily dressing of the surgical wound was maintained for 10 days using 2% povidone iodine. Systemic antibiotic (Ceftriaxone® 1000 mg i.m., Novartis Co., Sandoz, Switzerland) was administered at a dose of 1 gm/dog for 7 days. Skin sutures were removed 10 days following surgery.

During the study, dog’s general health condition was evaluated (behavior/body weight/appetite/oral function). Surgical site was evaluated for allergic or inflammatory reaction (edema, hyperemia or swelling).

#### Euthanasia and sampling

Dogs were humanely euthanized at 12 weeks using sodium pentobarbitone 200 mg (Eutha-naze®, Premier pharmaceutical Co., Bryanston, South Africa) at a dose of 2 ml/kg. Just following euthanasia, both mandibular halves were dissected, labelled, and sent for CBCT examination.

### Cone beam computed tomography (CBCT)

**Fig. 2:**
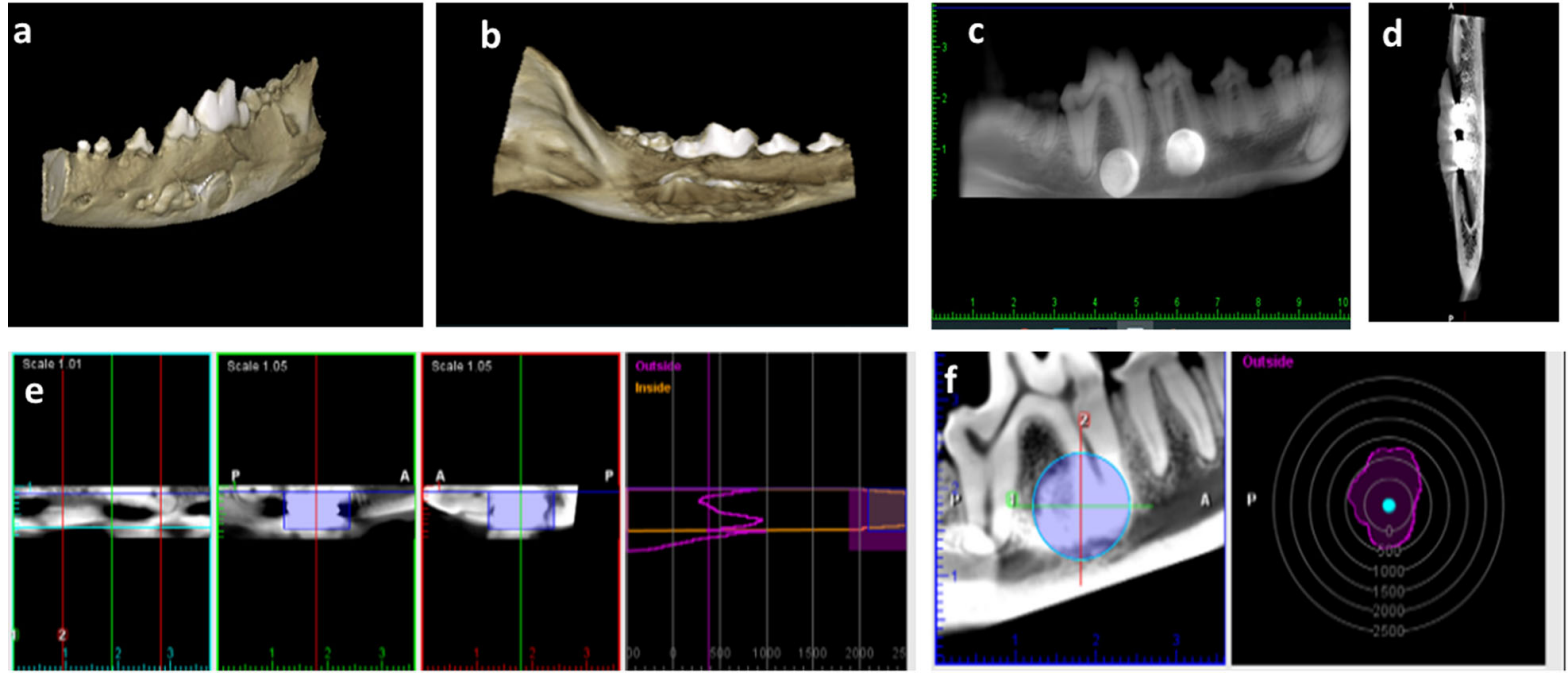
CBCT examination of mandibular defects demonstrating 3-D (**a**, **b**) and panoramic views (**c**–**f**) of the implanted alloys at different planes. Radiographic bone density was measured the verification tool by drawing a virtual circle in a constant area in sagittal and coronal views (**e**, **f**)

A Planmeca (Helsinki, Finland) CBCT machine was used with the following specifications 10 mAs, 86 kVp, 11.2 s exposure time. An 8 × 8 cm field of view with a dimension of 534 × 534 × 534 and 150 µm voxel size was used. Three dimensional and panoramic views were captured as general views outlining and localizing the implanted alloys as well as the surrounding mandibular bones. Cross-sectional views were taken from buccal to lingual sides through the defects to evaluate bone healing and integration of the alloy with the surrounding bones, inferior alveolar canal, and biomineralization. The incorporated software program of the machine (Romexis1version 4.4.1.R, Planmeca, Helsinki, Finland) was used to quantify bone density measurement. Radiographic bone density surrounding the implants was recorded using the verification tool in the Romaxis 1 software by drawing a virtual circle in a constant area for sagittal and coronal views. Ten radiodensity (Hounsfield; HU) readings were recorded for each mandibular defect, and the mean bone density values were calculated and expressed as bone density in Hounsfield (HU) units (Fig. 2).

### SEM/EDX examination

After CBCT examination, the implants were carefully removed from five dogs and mechanically cleaned using ultrasonic bath for 10 min. Implants were dried at room temperature, labelled and sent for SEM/EDX examination.

### Histological examination

Mandibular bone defects, including the defect site as the surrounding tissue, were harvested, labelled, and sent for blind histological evaluation. After being washed, samples were fixed in a 10% calcium formal solution for 48 h, decalcified for 4–5 weeks using a 10% EDTA solution [19]. Following decalcification, samples underwent series dehydration, clearing with xylene, and embedding in paraffin blocks. Paraffin blocks were sectioned at 5 μm thick for Hematoxylin and Eosin (H&E) and Masson Trichrome (MT) staining (H&E: ab245880, MT: ab150686; Abcam, Egypt Distributor; GeneTech Company). Bone samples were examined under a light microscope equipped with a digital camera (Leica DM300 Microsystems, Inc., Switzerland).

### Histomorphometric analysis

Histomorphometric analysis was conducted to measure the percentage of bone area and the percentage of mature bone area using Fiji Image J, an image analysis program. The trainable Weka segmentation method and the bone J plugin were used [20]. Image analysis was conducted on pictures obtained with a magnification of X100. Each sample was measured in five fields, and the mean was calculated. Bone area percentage was directly quantified in H&E sections whereas the area percentage of matured bone area was assessed in MT sections.

### Quantitative Real-Time PCR analysis for gene expression of osteopontin and osteocalcin

Bone samples were collected from all groups and homogenized. Total RNA was extracted using Direct-zol RNA Miniprep Plus (Cat#R2072, ZYMO RESEARCH CORP. USA). The Beckman dual spectrophotometer (USA) was used to evaluate the quantity and purity of the extracted RNA. The reverse transcription of extracted RNA was conducted using a One-Step RT-PCR reagent (Cat#12594100, Thermo Fisher Scientific, Waltham, MA, USA) and subsequently followed by PCR 48-well tray. Thermal profile was conducted using Step-One instrument (Applied Biosystem, USA) in the following manner: The cDNA synthesis process involves a single cycle of 45 °C for 15 min, followed by 10 min at 95 °C to inactivate the reverse transcriptase enzyme. This is followed by 40 cycles of PCR amplification. Each cycle was maintained for a duration of 10 s at 95 °C, 30 s at 60 °C, and 30 s at 72 °C. Data were expressed in Cycle threshold (Ct) for the target genes and housekeeping gene GAPDH following the RT-PCR run. Osteopontin and osteocalcin were normalized for variation in the expression of each target gene (Table 1) using the ΔΔCt method, with the mean critical threshold (CT) expression value of the GAPDH housekeeping gene as the reference. The 2-∆∆Ct method is used to calculate the relative quantitation (RQ) of each target gene.

**Table 1:** Forward and reverse primers and the housekeeping gene used for osteopontin and osteocalcin RT-PCR analysis

Gene	Forward Primer	Reverse Primer	GenBank Accession No.
**GAPDH**	TGGTGAAGACGCCAGTGGA	GCACCGTCAAGGCTGAGAAC	NM_002046
**Osteocalcin**	CTCACACTCCTCGCCCTATTG	GCCTGGGTCTCTTCACTACCT	BC113434
**Osteopontin**	CAGTTGTCCCCACAGTAGACAC	GTGATGTCCTCGTCTGTAGCATC	J04765

### Statistical analysis

Numerical data were presented as the mean ± standard deviation. Kolmogorov-Smirnov test was employed to ensure normality of distribution. A one-way analysis of variance (ANOVA) test was implemented to compare different groups followed by Tukey’s post hoc test. The significance level was established at *p* < 0.05. Data were analysed using SPSS (Statistical Package for Scientific Studies, SPSS, Inc., Chicago, IL, USA) software version 22.

## Results

### Clinical evaluation

Clinical evaluation demonstrated that healing was eventual in all dogs. None of the dogs demonstrated any signs of local or systemic adverse, allergic, nor inflammatory reaction. All skin wounds were healed within 7–10 days following surgery. Dogs maintained normal appetite, normal masticatory function and body weight remained stable throughout the study.

### CBCT examination

**Fig. 3:**
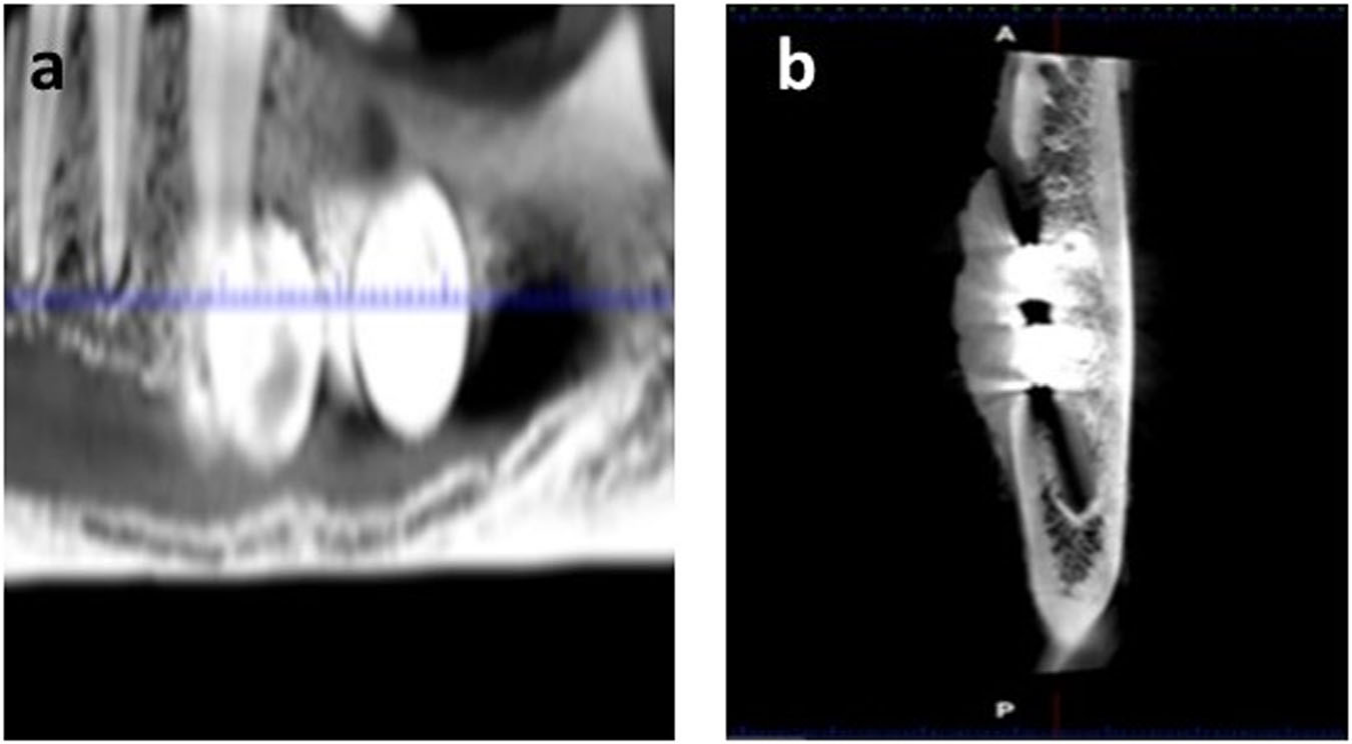
Panoramic (**a**) and crossictional (**b**) cone beam computed tomography of mandibular defects demonstrating the presence of slight hazes around the implanted alloys at 12 weeks following implantation

CBCT examination confirmed proper implant positioning within the induced mandibular defects. All implanted defects demonstrated the presence of slight hazes around the implanted alloys (Fig. 3). The mean radiographic bone density of mandibular bone around the implanted alloys demonstrated statistically significant differences among all groups (*p* = 0.000) (Table 2).

**Table 2:** The mean radiographic bone density of mandibular bone around the implanted alloys (Hu unit) as determined by CBCT examination

Group	Mean ± SD	Std. Error	Minimum	Maximum	95% Confidence Interval for Mean		*P* value
					Lower Bound	Upper Bound	
M	133.6 ± 23.3	9.5	100.0	170.0	109.1	158.1	0.000*
M0	266.0 ± 17.4	7.1	240.0	290.0	247.7	284.3	
M1	510.6 ± 37.2	15.2	450.0	557.8	471.5	549.6	
M2	658.2 ± 24.3	9.9	625.8	690.0	632.6	683.7	
M3	724.6 ± 41.1	16.8	690.0	800.0	681.4	767.7	

*Significance level *P* < 0.05

### SEM-EDX evaluation

**Fig. 4:**
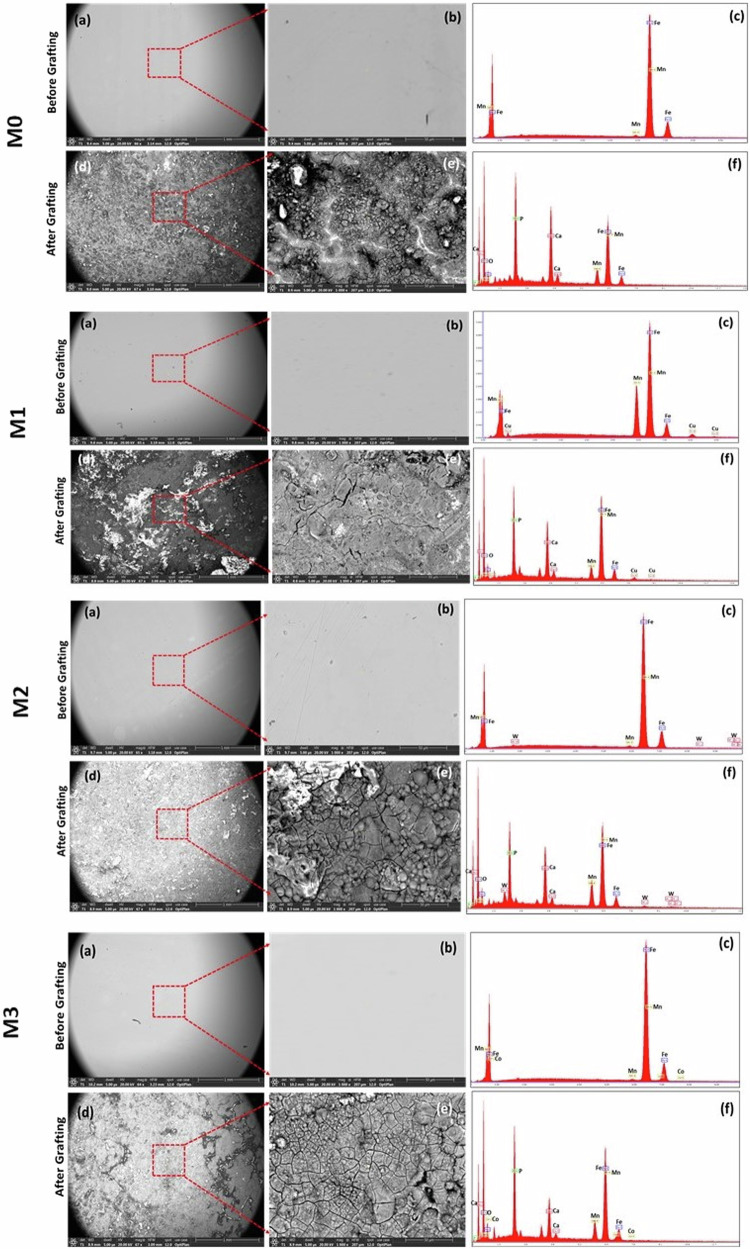
SEM-EDX at low and high magnifications and BSE analysis of different alloys before (**a**–**c**) and after (**d**–**f**) implantation. M0 alloy demonstrated the presence of Ca, P, and O in association with Fe and Mn. M1 alloy demonstrated the presence of Ca, P, and O in association with Fe, Mn and Cu. M2 alloy demonstrated the presence of Ca, P, and O in association with Fe, Mn and W. M3 alloy demonstrated the presence of Ca, P, and O in association with Fe, Mn and Co

Microstructure properties and corrosive behavior demonstrated that before in vivo implantation, all alloys exhibited an almost solid solution without any notable phases in microstructure as shown in Fig. 4a–c.

While after in vivo implantation, the SEM-EDX alloy elemental analysis displayed new features with the presence of other elements, mainly calcium (Ca), phosphorus (P), and oxygen (O), in addition to the main elements forming each alloy (Fig. 4d–f).

TEAM software analysis of the detected x-ray spectrums and calculate. The mean values of x-ray spectrums of elements detected in different alloys before and after grafting is demonstrated in Table 3.

**Table 3:** Elemental analysis of different alloys before and after grafting using Energy Dispersive X-ray Spectroscopy (EDX)

Alloy	Elemental analysis (mean)							
	Before grafting				After grafting			
	Element	Wt. %	At. %	Er. %	Element	Wt. %	At. %	Er. %
**M0**	**Mn**	31.26	31.61	5.14	**Mn**	7.55	3.55	9.06
	**Fe**	68.74	68.39	4.23	**Fe**	28.14	13.00	6.65
	-	-	-	-	**O**	41.20	66.45	6.04
	-	-	-	-	**P**	11.21	9.34	5.90
	-	-	-	-	**Ca**	11.90	7.66	5.39
**M1**	**Mn**	29.87	30.35	4.80	**Mn**	6.28	3.13	8.84
	**Fe**	66.31	66.29	4.34	**Fe**	35.71	17.52	5.50
	**Cu**	3.82	3.36	8.07	**Cu**	2.42	1.04	6.77
	-	-	-	-	**O**	37.35	63.93	3.06
	-	-	-	-	**P**	9.56	8.45	5.71
	-	-	-	-	**Ca**	8.68	5.93	5.58
**M2**	**Mn**	33.19	34.04	3.99	**Mn**	12.65	6.89	7.11
	**Fe**	64.74	65.32	3.81	**Fe**	33.50	17.94	5.98
	**W**	2.07	0.64	9.94	**W**	4.25	0.69	8.74
	-	-	-	-	**O**	31.61	59.10	4.86
	-	-	-	-	**P**	8.88	8.58	5.72
	-	-	-	-	**Ca**	9.11	6.80	5.28
**M3**	**Mn**	28.97	29.34	3.72	**Mn**	9.19	4.69	7.63
	**Fe**	69.10	68.84	4.20	**Fe**	36.74	18.43	5.53
	**Co**	1.93	1.82	10.06	**Co**	0.73	0.35	14.71
	-	-	-	-	**O**	34.93	61.19	6.06
	-	-	-	-	**P**	12.00	10.86	6.51
	-	-	-	-	**Ca**	6.41	4.48	7.30

*At %* Atomic percent, *Er%* Error percent, *Wt %* weight percent

### Histological examination

**Fig. 5:**
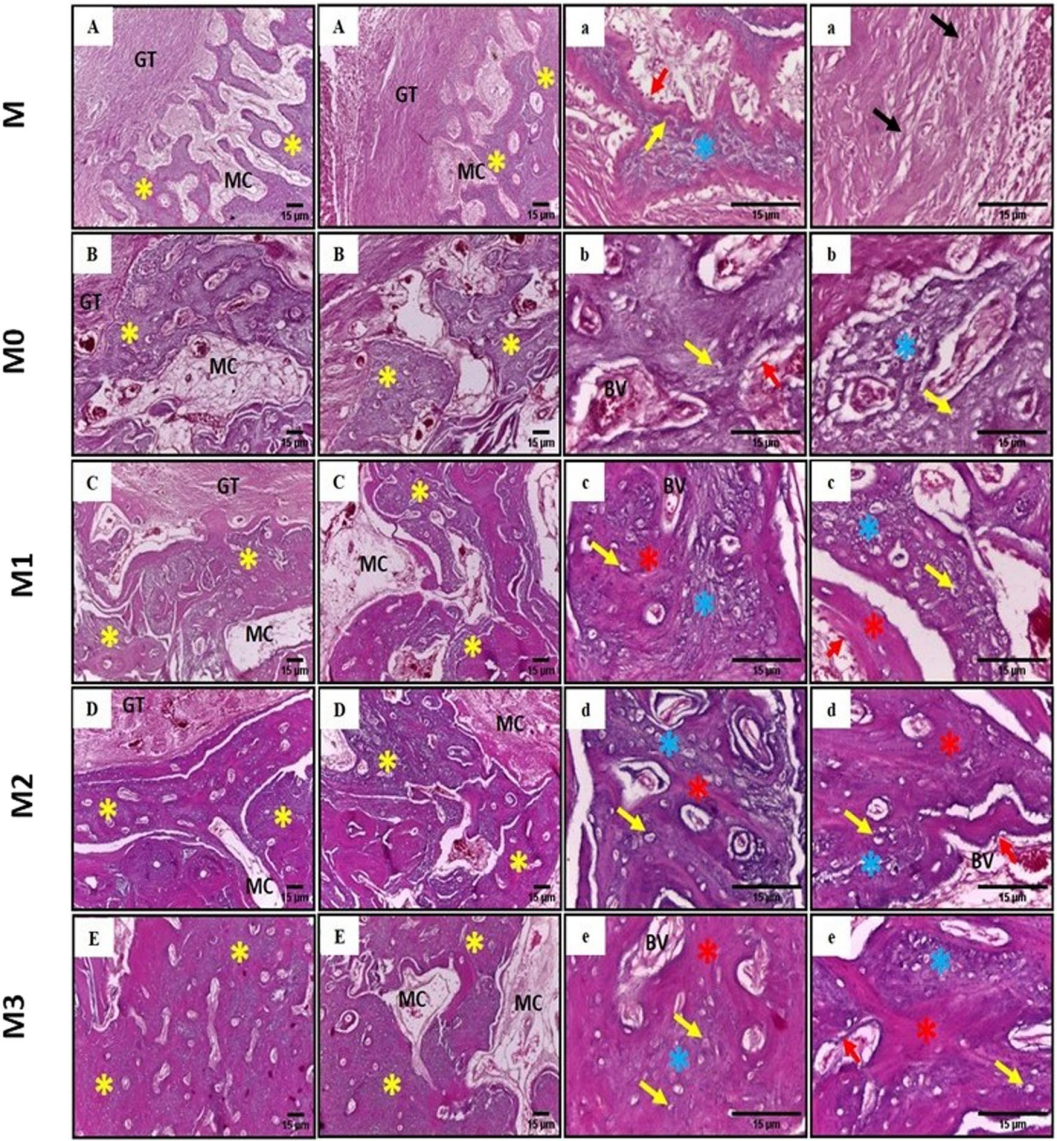
Photomicrographs of the mandibular bony defects (**A**) M control group, (**B**) M0, (**C**) M1, (**D**) M2 and (**E**) M3 showing; areas of newly formed bone (yellow asterisks), fibrocellular marrow cavities (MC) and granulation tissue (GT) (H&E X100) (Scale bar = 15 μm), while (a, b, c, d, e) are higher magnification of each experimental group represent the newly formed woven bone (blue asterisks), randomly distributed entrapped osteocytes (yellow arrows), osteoblastic cells (red arrows), blood vessels (BV), areas of lamellar bone (red asterisks), and well-arranged fine collagen fibrils (black arrows) (H&E X400) (scale bar = 15 μm)

Histological sections of control mandibular bone (Group M) stained with H&E demonstrated thin, irregularly arranged, newly formed bone trabeculae encircling large marrow cavities (Fig. 5A). Well-arranged fine collagen fibrils were spotted filling most of the defect area (Fig. 5A). In comparison, defects implanted with biodegradable metal alloys (Group M0, M1, M2, M3) demonstrated thicker, recently formed bone trabeculae made of woven bone that were enclosed by extremely fibrous connective tissue and mixed with areas of lamellar bone (Fig. 5B–E). Higher magnification of the newly formed bone trabeculae revealed numerous randomly distributed large-sized osteocytes and the marrow cavities were bordered by osteoblastic cells (Fig. 5A–E). Group M1, M2, and M3 possessed higher degree of development and organization in lamellar bone structure, characterized by intricate interlacing patterns and thin regions of woven bone that enclose several narrow marrow cavities compared to M0 group (Fig. 5C–E). Occasionally, the newly formed bone trabeculae formed Haversian systems by arranging themselves in a circular pattern around marrow cavity (Fig. 5C–E). Additionally, endothelial-lined blood vessels were seen (Fig. 5B–E).

**Fig. 6:**
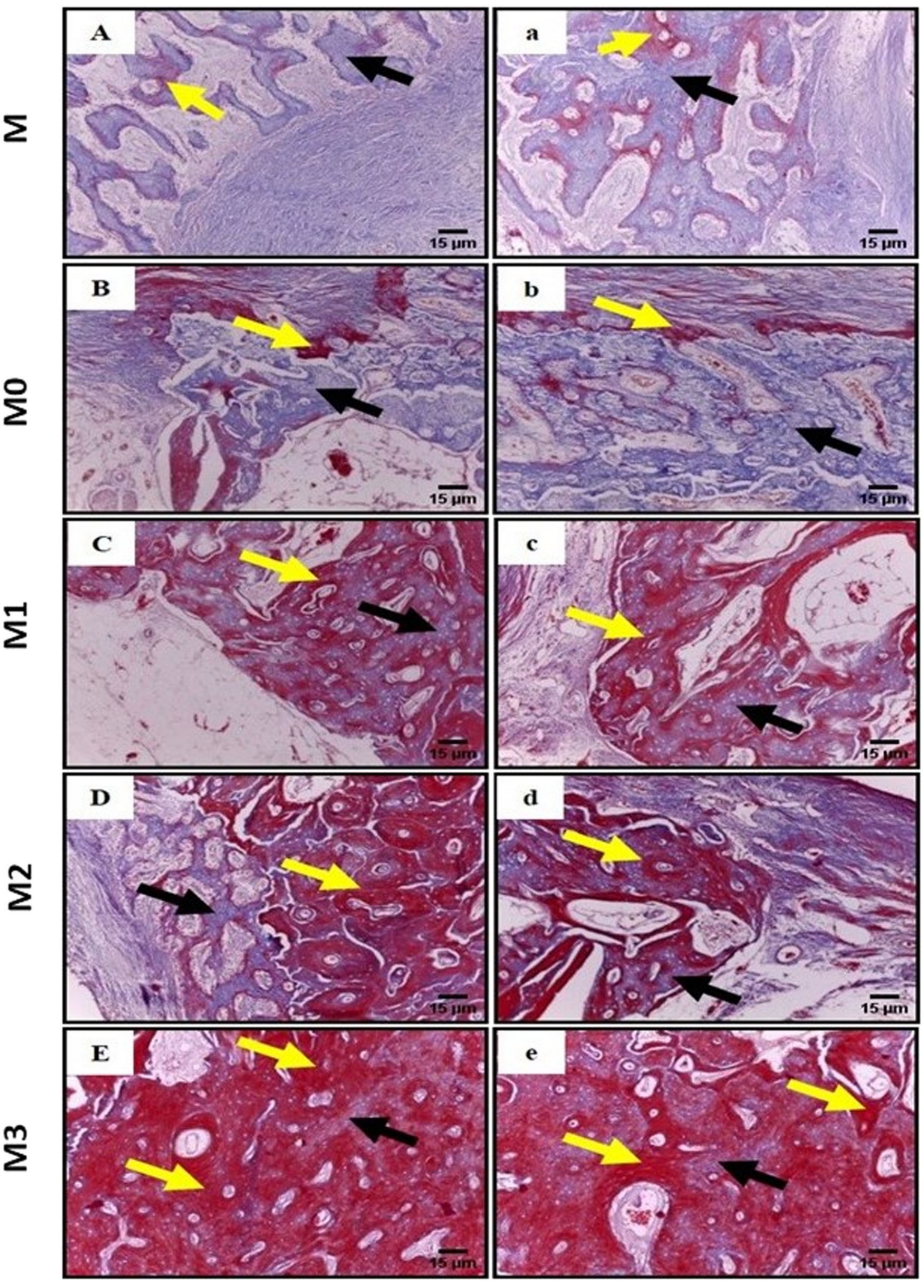
Photomicrograph of the mandibular bony defects (**A**) control group, (**B**) M0, (**C**) M1, (**D**) M2 and (**E**) M3 showing; collagen fibers of the newly produced woven bone are denoted by the blue color (black arrows). Additionally, areas of lamellar bone are indicated with a red color (yellow arrows) (MTx100) (Scale bar = 15 μm)

Bone sections stained with MT demonstrated varying levels of bone maturation. In control (M) and M0 groups, the presence of new collagen fibres within the newly formed bone trabeculae was evident. These collagen fibres were stained blue, indicating an immature woven bone. Small regions of lamellar bone, represented by a red color, were observed in these sections (Fig. 5A, B). Bone defects in M1 group exhibited a notable intermingling of woven and lamellar bone regions (Fig. 6C). Bone defects in the M2 and M3 groups exhibited a significant increase in the red color (Fig. 6D, E).

### Histomorphometric examination

#### Bone area percentage

Quantitative evaluation of bone area percentage demonstrated statistically significant highest bone area percentage in Group M3 compared to M2, M1, M0, and M group. A statistically significant increase in bone area percentage was recorded in M2 group compared to M1, M0, and M groups. A significantly increased bone area percent was recorded in M1 compared to control M group and also between M0 and M group. No statistically significant differences were recorded in bone area percentage between M1 and M0 groups (Tables 4 and 5).

**Table 4:** Mean bone area percentage and area percentage of mature bone (as demonstrated by Masson Trichrome staining) of mandibular bone defects implanted with different alloys

Parameter	Group	Mean ± SD	Std. Error	95% Confidence interval for mean		*P*
				Lower limit	Upper limit	
Bone area percent	M	28.19 ± 3.57^d^	1.46	24.45	31.94	0.000*
	M0	40.82 ± 3.93^c^	1.60	36.70	44.95	
	M1	48.85 ± 4.47^c^	1.82	44.17	53.54	
	M2	59.16 ± 4.99^b^	2.04	53.92	64.40	
	M3	71.61 ± 9.93^a^	4.05	61.19	82.03	
Area percent of mature bone (Masson)	M	29.33 ± 1.41^e^	0.57	26.71	31.95	0.000*
	M0	38.85 ± 3.33^d^	1.36	36.23	41.47	
	M1	51.82 ± 2.58^c^	1.05	49.20	54.44	
	M2	60.09 ± 4.42^b^	1.81	57.47	62.71	
	M3	80.86 ± 3.04^a^	1.24	78.24	83.48	

Significance level *P* < 0.05, *significant

Means with different superscript letters are significantly different

**Table 5:** Differences in bone area percent and area percent of mature bone of different groups as demonstrated by Tukey’s post hoc test

Parameter	Difference of levels		95% Confidence interval		Adjusted *P*-value
			Lower limit	Upper limit	
Bone area percent area	**M0**	**M**	2.70	22.56	0.008*
	**M1**	**M**	10.73	30.59	0.000*
		**M0**	−1.90	17.96	0.156
	**M2**	**M**	21.04	40.89	0.000*
		**M0**	8.41	28.26	0.000*
		**M1**	0.38	20.23	0.039*
	**M3**	**M**	33.49	53.34	0.000*
		**M0**	20.86	40.71	0.000*
		**M1**	12.83	32.68	0.000*
		**M2**	2.52	22.37	0.009*
Percent of mature bone	**M0**	**M**	4.24	14.80	0.000*
	**M1**	**M**	17.21	27.77	0.000*
		**M0**	7.70	18.26	0.000*
	**M2**	**M**	25.48	36.04)	0.000*
		**M0**	15.96	26.52	0.000*
		**M1**	2.99	13.55	0.001*
	**M3**	**M**	46.25	56.81	0.000*
		**M0**	36.74	47.30	0.000*
		**M1**	23.76	34.32	0.000*
		**M2**	15.50	26.05	0.000*

*Significance level *P* < 0.05

#### Area percent of mature bone

A statistically significant higher area percent of mature bone was detected in the M3 group as compared to other groups (M2, M1, M0, and M groups). A significantly higher area percent was also recorded in M2 group compared to M1, M0, and M groups. A significantly higher value was also recorded in M1 as compared to M0 and M groups and in M0 as compared to the M group. (Tables 4 and 5).

### Gene expression using qRT-PCR for osteopontin and osteocalcin

A statistically significant increase of mean osteopontin and osteocalcin gene expression was recorded in the M3 group compared to other groups (M2, M1, M0, and M groups). A significantly higher mean was also recorded in M2 group compared to M1, M0, and M groups. A significantly higher value was also recorded in M1 as compared to M0 and M groups. Although the difference between M0 and M groups was not statistically significant for osteopontin gene expression, it was significantly higher in osteocalcin gene expression. (Tables 6 and 7).

**Table 6:** Gene expression of osteopontin and osteocalcin in mandibular bone defects implanted with different alloys

Parameter	Group	Mean	Std. Error	95% Confidence interval for mean		*P*
				Upper limit	Lower limit	
Osteopontin	M	1.00 ± 0.00^d^	0.00	1.00	1.00	0.000*
	M0	1.09 ± 0.07^d^	0.03	1.02	1.17	
	M1	1.79 ± 0.12^c^	0.05	1.66	1.91	
	M2	2.85 ± 0.11^b^	0.04	2.74	2.97	
	M3	4.55 ± 0.16^a^	0.064	4.38	4.71	
Osteocalcin	M	1.00 ± 0.00^e^	0.00	1.00	1.00	0.000*
	M0	1.16 ± 0.07^d^	0.03	1.08	1.23	
	M1	1.69 ± 0.11^c^	0.05	1.56	1.80	
	M2	2.41 ± 0.08^b^	0.03	2.34	2.50	
	M3	3.48 ± 0.08^a^	0.032	3.40	3.56	

*Significance level *P* < 0.05

Means with different superscript letters are significantly different

**Table 7:** Differences in gene expression of osteopontin and osteocalcin among different groups as demonstrated by Tukey’s post hoc test

Parameter	Difference of levels		95% Confidence interval		Adjusted *P*-value
			Lower limit	Upper limit	
Osteopontin	**M0**	**M**	−0.08	0.28	0.544
	**M1**	**M**	0.61	0.97	0.000*
		**M0**	0.51	0.87	0.000*
	**M2**	**M**	1.67	2.03	0.000*
		**M0**	1.58	1.94	0.000*
		**M1**	0.88	1.25	0.000*
	**M3**	**M**	3.37	3.73	0.000*
		**M0**	3.27	3.63	0.000*
		**M1**	2.58	2.94	0.000*
		**M2**	1.51	1.87	0.000*
Osteocalcin	**M0**	**M**	0.03	0.29	0.014*
	**M1**	**M**	0.55	0.81	0.000*
		**M0**	0.39	0.66	0.000*
	**M2**	**M**	1.29	1.55	0.000*
		**M0**	1.13	1.40	0.000*
		**M1**	0.60	0.87	0.000*
	**M3**	**M**	2.35	2.61	0.000*
		**M0**	2.20	2.46	0.000*
		**M1**	1.67	1.93	0.000*
		**M2**	0.93	1.20	0.000*

*Significance level *P* < 0.05

## Discussion

The present study demonstrated that combining Cu/W/Co in Fe-Mn nanostructured biodegradable alloys exhibited promoted bone formation with improved biocompatibility and osseointegration of the alloys and surrounding bone in dog model.

Our preliminary in vitro studies have concluded that the novel nanostructured biodegradable Fe–Mn–Cu, Fe–Mn–W, and Fe–Mn–Co metal alloys demonestrated good biocompatibility on oral epithelial cell lines with the enhancement of cell proliferation in a time-dependent manner that favors bone regeneration. In addition, the three alloys demonestrated anticancer activity against MG-63: osteosarcoma cell line [21].

Before being used in clinical practice, bone implants must be validated for bio-functionality, biocompatibility, biodegradability, osteointegrative, osteoconductive, and osteoinductive properties using animal models [22]. The dog presents an ideal pre-clinical model for studying bone healing and regeneration because its bone macro-and microstructure, density, and turnover are all very similar to human bone [23, 24].

Biocompatibility is crucial for the successful outcome, where precise alignment and fixation is of the utmost importance for proper healing and function. Biocompatibility minimizes the risk of adverse allergic reactions especially for patients with sensitivities or allergic tendencies against conventional implants [3, 25].

One of the key advantages of using biodegradable metal alloys is that they gradually dissolve in the body, stimulating new bone tissue forms providing support and strength to the healing bone structure. This can help to reduce the risk of inflammation, minimize the chance of rejection, and promote faster recovery, which is of the utmost importance in complex dental procedures involving tooth extraction, implant placement and bone grafting [2, 5].

Scanning electron microscopy and EDX examination before and after implantation of the alloys revealed signs of degradation on the sample surfaces. It is difficult to precisely determine the small losses in volume or mass of the implanted alloys where degradation products might be unintentionally added to the volume of the metal following implantation [5]. Even though weighing is an accurate technique that can be employed to alloys before and after implantation, it may not be possible to fully eliminate the degradation products while cleaning the retrievals [5].

The faint hazes recorded around the implanted alloys in CBCT scan could be attributed to degradation by-products (mainly oxygen) or are associated with an artifact. Metallic materials can introduce artifacts in μCT scans, where beam hardening posing a challenge in analysing and quantifying areas of corrosion, particularly in regions with minimal corrosion and low density [26].

Despite that the current in vivo model is a relatively short-term study (3 months) to evaluate biodegradability of the alloys, remarkable slow degradation process was recorded in different alloys as confirmed by SEM-EDX analysis. It is crucial to highlight that no precise records have been published to quantify the amount of material loss caused by degradation. Findings from previous studies on pure iron stents detected degradation products after one month [6] while Krause et al., reported slow degradation of Fe pins implanted into rat’s femur over a one-year [5].

Interestingly, the degradation rate of Fe-based materials in the in vitro experiments and in vivo models revealed noticeable disparities. The in vitro degradation rates of pure Fe and Fe-based alloys as observed through immersion tests in physiological media (SPF and Hank’s solution) suggest that Fe–Mn alloys degrade notably faster than pure Fe [5, 27]. It is important to consider the electrochemical processes occurring during the in vivo degradation of Fe-based alloys and the in vivo environmental conditions present where the alloys are implanted [5].

The anodic partial reaction of metal oxidation (Fe → Fe^2+^ + 2e^−^) proceed rapidly in media.

In oxygen-containing aqueous solution (pH 4–10), the reduction of dissolved oxygen occurs in cathodic partial reaction (H_2_O + 1/2O_2_ + 2e^−^ → 2OH^−^.

The availability of oxygen in in vivo models affects the degradation rate of Fe-based implants. This argument explains the variations in degradation rates of Fe implants at different in vivo studies. Pure Fe stents implanted within the blood vessels degraded faster than Fe pins. Circulating blood provides a continuous supply of oxygen, resulting in a faster corrosion reaction on metal surfaces. The oxidation process usually begins in the outer layers due to an oxygen gradient, advancing towards the metal surface over time. In vitro [27] and in vivo [6] studies have confirmed the formation of Fe-oxide degradation products. Examination using SEM revealed a layered structure of these degradation products. The first layer, closest to the metal, consists of oxidized components such as Fe, Mn, Pd, and O. Above this layer, a second layer contains not only Fe, Mn, and O but also elements originating from biological organisms, including P, Ca, Na, and K [5].

In the current study, histologic examination was crucial to assess tissue compatibility and inflammatory reaction induced by the implanted alloys. Biocompatibility was indicated by the absence of an inflammatory response and the formation of new mature bone tissue around the implanted alloys. The bone’s overall recovery process was eventual, and none of the alloys exhibited signs of infection nor tissue rejection.

Histologic examination of control empty defects demonstrated expressing the least late osteogenic bone marker (osteopontin and osteonectin). While Fe-Mn based alloys demonstrated favorable osteogenic potential. In vivo studies demonstrated that iron alone was not sufficient to promote bone regeneration due to its slow degradation rate [28]. Also in vitro experiment conducted using MG-63 osteosarcoma cells demonstrated that iron ions decreased cell proliferation rates [29]. The addition of alloying elements such as 20–35% manganese can substantially increase the degradation rates, with enhanced biological activity, cell compatibility and corrosion rate compared to pure iron [4, 30].

Manganese plays a pivotal role in protein synthesis in bone tissues [31]. Manganese ions activate integrin and positively affect osteoblast attachment, survival, and proliferation [32, 33]. Biomaterials containing manganese ions exhibited elevated levels of osteogenic gene expression and increased collagen deposition [34, 35]. Manganese-containing bioceramics demonstrated efficient scavenging of superoxide anions and hydrogen peroxide free radicals, as well as inhibition of osteoclast activity and promoting osteogenic differentiation. Mn converts superoxide anion into oxygen and water, immediately limit the build-up of hydrogen peroxide, inhibiting osteoclast formation and promoting osteogenic differentiation [36].

In the present study, incorporating Cu into Fe-Mn alloy (Group M1) revealed an increase in amount and maturation of newly formed bone and osteogenic gene expression compared to M and M0 groups. Copper can stimulate the growth of osteoblasts, enhancing osteogenesis with faster healing of bone defects [29, 30]. Cu-based alloys results in reduction in Adenosine 5‘-monophosphate (AMP)-activated protein kinase (AMPK) and p-AMPK. This mechanism suggests that copper ions can activate the mammalian target of rapamycin (mTOR) signaling pathway by suppressing AMPK phosphorylation leading to stimulation of osteoblast proliferation [37]. The presence of copper in bioactive glass can increase the process of lipid peroxidation, which is linked to the growth improvement of human osteosarcoma cells that resemble osteoblasts [38]. Incorporating copper into different biomaterials demonstrated beneficial effects on bone formation, evidenced by the increased activity of the alkaline phosphatase enzyme, which is involved in the early-stage differentiation of bone MSCs. Significant increase in the expression of various genes related to bone formation, including Runx2, alkaline phosphatase, collagen I, osteocalcin, osteopontin, and bone morphogenetic protein, was promoted [37, 39, 40]. These results coincide with our results, suggesting that copper can promote the maturation of osteoblasts from osteogenic precursor cells. Copper ions are known to affect osteogenic differentiation through a process often associated with the modulation of inflammatory responses, where copper regulates a beneficial inflammatory milieu that promotes osteogenesis [41]. Releasing copper from bioceramic surface can activate specific signaling pathways (CTR1 and ATP7A) in macrophages, leading to polarization of these cells towards a pro-inflammatory M1 phenotype. This activation is supported by the increased expression of M1 surface markers (CD86 and CD11c) and pro-inflammatory cytokines (TNF-α, IL-6, iNOS, and IL-1β) [42, 43].

Tungsten was incorporated to biodegradable iron alloy due to its exceptional biocompatibility and slower breakdown rate compared to iron [7, 44]. Tungsten demonstrated promising osteogenic potential in bone regeneration, evidenced by increased bone formation and maturation in addition to increased osteocalcin and osteopontin gene expression. Our results disagree with those of Bolt et al., who demonstrated that tungsten inhibited the osteogenesis of MSCs in vitro and correlated with an increase in the content of adipocytes in the bone [13]. Despite their in vitro results, the in vivo results of their study presented no evidence indicating that tungsten impeded the process of osteogenesis.

Although bone formation was evident in all groups, the histological and histomorphometric analysis of incorporating cobalt to Fe-Mn alloy (Group M3) revealed thick, dense bone trabeculae with the highest bone area % of newly formed bone compared to other groups. In vivo studies demonstrated that in presence of Co^2+^, the utilization of cobalt chloride facilitates the formation of vascularized bone both in ectopic and orthotopic areas [16]. Additionally, Co^2+^ has been shown to promote angiogenesis by establishing a hypoxic environment through activating hypoxia-inducing factor (HIF) signalling [25]. The addition of Co^2+^ ions to scaffolds, such as bioglass, clearly induces hypoxia and can improve the secretion of vascular endothelial growth factor (VEGF), production of HIF-1α, and expression of bone-related genes in BMSCs [15]. In agreement with previous studies, expression of osteocalcin and osteopontin gene is stimulated when incorporating cobalt into alloys. Jiménez-Holguín et al., reported an increase in gene expression of early and late osteogenic markers (alkaline phosphatase, RUNX2, and osteocalcin) as well as the VEGF in the presence of Co^2+^ [45]. Moreover, addition of cobalt enhances hydroxyapatite-based scaffolds’ ability to promote bone formation. The cobalt hydroxyapatite derivative, which contains 1.25% cobalt, showed increased attachment and proliferation of MG-63 cells. Their mineralization and osteogenic marker expression regarding calcium deposition and alkaline phosphatase activity were elevated [14]. However, one of the most debatable aspects of cobalt-doped bioactive glasses is their potential detrimental impact on the viability and differentiation of various cell types, such as osteoblasts and osteoprogenitor cells [46]. Hoppe et al. has shown a clear correlation between the cytotoxicity and the concentration of Co^2+^ ions in the culture medium. They have identified a safety range of 2 to 12 ppm [47].

In conclusion, the use of biodegradable metal alloys in maxillofacial surgery represents a promising advancement in the field of implantology. Overall, incorporating Cu/W/Co into Fe-Mn provided improved tissue regeneration, good balance of strength, corrosion resistance, enhanced biocompatibility, and environmental sustainability making it suitable for use in various orthopedic and dental applications. By providing a solution to the limitations of traditional non-biodegradable implants, biodegradable metal alloys have the potential to improve patient outcomes, reduce the need for additional surgeries. However, there are still challenges to overcome in the widespread adoption of biodegradable metal alloys in maxillofacial surgery. The long-term biocompatibility and degradation rates need extensive research to ensure predictable outcomes and patient safety.
